# Dynamics of human resource department ecosystem in developing human resource role: An ecosystem perspective

**DOI:** 10.1371/journal.pone.0295544

**Published:** 2023-12-14

**Authors:** Henndy Ginting, Veronica Afridita Khristiningrum, Aurik Gustomo, Anggara Wisesa, Jumadil Saputra

**Affiliations:** 1 School of Business and Management, Institut Teknologi Bandung, Bandung, Indonesia; 2 Doctoral Program of Science in Management, School of Business and Management, Institut Teknologi Bandung, Bandung, Indonesia; 3 Universiti Malaysia Terengganu, Terengganu, Malaysia; National University of Sciences and Technology, PAKISTAN

## Abstract

Over the last decade, management scholars have paid increasing attention to ecosystems. The ecosystem approach has recently received much attention in business and innovation studies as a comprehensive way of understanding multi-aspect environments. This exploratory study aims to investigate the dynamics of human resource department ecosystem (HRDE) and the role of HRDE in shaping the human resource business partner role. This study employed a mixed-methods approach, following an exploratory sequential design. First, a conceptual model was developed based on qualitative data collected from expert interviews and analyzed through grounded theory. This stage uncovered eight actors and four factors, further organized into three layers of the ecosystem and hypothesis paths. Then, the structural model was measured and validated using PLS-SEM. This study is unique in applying the HRDE to the HR role’s development to deepen our understanding of how a human resource business partner role is shaped by actors’ interactions within and between ecosystem layers (micro, meso, and macro). The results revealed actors and factors supporting the HRDE in developing the HR role from the micro to macro layers of the ecosystem. The results suggest that the macro, meso, and micro layers of the HRDE positively impact the human resource business partner role’s development.

## Introduction

Over the last decade, management scholars have paid increasing attention to ecosystems [1–3]. Management scholars have studied ecosystems since [4] introduced the business ecosystem concept, which was inspired by the concept of biological ecosystems. The ecosystem approach has recently received much attention in business and innovation studies as a comprehensive way of understanding multi-aspect environments. The ecosystem concept has become popular in depicting cross-organizational collaboration [4–6]. The ecosystem, origins in ecology and biology, refers to a collection of organisms, their environment, and their interactions in a specific geographic area [7]. In natural or humanistic contexts, it can be regarded as a self-organizing holarctic open system [8]. Adopting this approach in various socioeconomic domains such as business, innovation and entrepreneurship is one of the most valuable and effective strategies to study and acquire a thorough understanding of these domains [9,10].

Disruption in human resource management (HRM) practices necessitates mutual adjustment processes within the organization that seek to address these changes properly, overcome tensions, and fit strategic needs. To be able to perform strategically, it is a necessity to develop a human resource (HR) role as a business partner (BP) for the organization [11]. Furthermore, HR needs to be innovative, breaking the traditional pattern of only personnel administration. In developing a strategic HR role, the HR department could not stand alone, acknowledging its limited resources. Accordingly, collaborating with multiple stakeholders across organizational boundaries must be acknowledged to manage organizational complexity and dynamics more effectively [12]. Collaboration is more common than ever in the environment of today, in part because people need to cooperate across corporate boundaries [13]. HR departments need to examine maintaining as many networks as possible and that relationships, partnerships, networks, alliances, and collaborations are crucial, especially for thriving in this rapidly evolving world [4,14]. The network could be firmly rooted within the HR department, inside the organization, or the external environment outside the organization, which becomes the ecosystem boundaries in this research.

To mention a few, HR researchers have addressed research on the HR role [15–18]. Furthermore, the HR role has been studied through the lens of different theories, such as the institutional and contingency theory [16], resource-based theory [15,17], role theory [19], upper echelon theory [20]. Most of the works discussed influencing factors of the HR role [16,17,19,20], while some others discussed about HR role influences other variables, such as business outcomes [15], HRM effectiveness [18] and organizational performance [21]. Moreover, prior research found several internal and external factors influencing HR roles within a company such as top management [22,23], line manager [24,25], external stakeholders [26]. We thus feel the lack of a holistic perspective that provides insight into the various layers (macro, meso, and micro) and dynamics of the human resource department ecosystem (HRDE) that positively relates to HR role development. Accordingly, there seems to be a need for an integrative and holistic approach to an examination of the HRDE toward a larger number of actors and interactions. To the best of our knowledge, none of the articles discussed the dynamic collaboration of how actors and factors influence the development of HR role.

This exploratory study aims to examine the impact of the HRDE in developing HR role in consumer goods companies from the perspective of the managerial positions and related staffs of the companies through two steps: (1) developing a conceptual framework of required indicators; (2) exploring the causal correlations of the indicators and HR role. Therefore, our research questions are (1) *how does the HR department collaborate with actors at each interacting ecosystem level in developing HR roles*? and (2) *what is the relationship between each ecosystem level in developing HR roles*? This study contributes to theory by applying the ecosystem approach to deepen our understanding of how HRDE influences HR role. Moreover, an ecosystem view allows us to identify the internal and external actors and factors in developing an HR role in a holistic perspective and explain the collaboration between actors to have a goal, developing an HR BP role. To our knowledge, this approach has not been explored by previous research on HR role. As this study is of developing HR role through the ecosystem theoretical lens, not previously applied in HR role research, we use a sequential mixed method whereby a qualitative, grounded theory method is first used to identify emergent themes from semi-structured interviews with HR professionals, followed by a quantitative method (survey) based on those themes. A qualitative study aims to understand how actors and factors at each ecosystem level are involved in developing HR roles and a quantitative study empirically tested the relationship between HRDE to HR role.

## 1.Theoretical background

### 1.1. Ecosystem perspective in developing HR role

An ecosystem is defined as “A biological system composed of all the organisms found in a particular physical environment, interacting with it and each other. Also, in extended use: a complex system resembling this” [27]. The ecosystem concept can be defined as a multi-stakeholder structure of organizations that materializes a common value proposition [1,2,6,28]. Ecosystems have two distinctive features compared to other collaborative concepts: complementarity and interdependence coexist, and the system is not fully hierarchically controlled [2,6]. In addition, scientists have started to study the diversity of ecosystem targets [29] by categorizing them into several classes, such as business objectives aimed at creating economic advantage, innovation or competitive position. [4] differentiates between a biological system and a business equivalent, where firms seeking new innovations interact and exist in a specific business environment. An ecosystem can be made up of a variety of individuals that interact closely with one another both inside and outside of the cluster. Furthermore, each individual in the ecosystem ultimately shares in the fate of the network as a whole, much like a particular species does in a biological ecosystem [30]. While [31] emphasize the co-evolution of competition and collaboration in the business ecosystem, [6] also emphasizes the interdependencies between actors and the simultaneous presence of complementarities where the interdependencies could connect within a system-level architecture.

Recently, the rise of the ecosystem approach has touched the human resource management field. Some studies try to apply the ecosystem approach in different contexts [32–36]. For instance, [34] argue the importance of the ecosystem in the HRM practices in the context of the gig economy while in [33] discussed about labor platform ecosystem, [36] in international HR development consider the interdependencies among its actors involved through meta-synthesis analysis, while [35] argue that rugby market is a human capital ecosystem with many actors responsible for producing aspirational value.

The study by [12,37,38] and [5] are some of the few studies that discussed the ecosystem theme as the emergence approach in viewing a more complex human resource management. [12] wanted to see HR as a more complex ecosystem and claimed that “to address the challenges of alignment in contemporary organizations, we need to frame HR as a more complex ecosystem” (p. 225), while in [37] discussed about workforce ecosystem. [38] proposed a multi-level framework of the human resource management ecosystem in (con)temporary organizing from a complex adaptive systems perspective. [5] proposed a model of the entrepreneurial ecosystem in human resource management (HRM) in some HR activities, such as talent acquisition, learning and development, performance management and rewards, and retention. The ecosystem theory is suitable for application in developing HR roles due to the limitation of the HR department in achieving the business partner role, which aligns with the concept of the business ecosystem where “no single firm has all of the required specialized knowledge and managerial resources necessary for the whole system” [39]. Furthermore, the HR department is considered as a complex system where many stakeholders cooperate and collaborate with and influence its roles, and this also could be theorized as an ecosystem.

[40] added that the system boundary should be set initially to limit the analysis area within the ecosystem, which could be set by geographical scope, temporal scale, etc. Furthermore, an ecosystem approach is needed to understand the holistic dynamics of a complex system by taking various system levels (micro, meso, and macro) into account to see how resources are integrated [41,42] proposed three levels of a multi-level perspective of context in the service ecosystem: micro level; meso level, and macro level. The micro level represents the interactions between individual actors, meso level represents the network of relationships, and the macro level represents the broader institutional context [43]. In human resource management research, the multilevel theory [44] divides organization levels as the basis to form the micro-level, meso-level, and macro-level perspectives in addressing such phenomena. The organization levels mentioned are intra-individual, individual, unit, organization, and outside [45]. The study of Bronfenbrenner’s ecological systems theory [46] explains that the different environmental systems influence human development comprised of four layers of ecological environment types: microsystem, mesosystem, exosystem, and macrosystem. In identifying the network boundaries in developing the HR BP role, this study adopted the ecological environment layers and used the organization boundary in setting each ecosystem boundary where the micro level limits inside the HR department, the meso level limits inside the company, and the macro level limits outside the company. Moreover, combining [1] and [6], the business ecosystem has three characteristics, namely: 1) activities: specific activities done by the actors in creating value proposition; 2) actors: entities who undertake activities; and 3) interactions: interactions between actors in the ecosystem. These characteristics are embedded in each micro, meso, and macro ecosystem level. Therefore, the ecosystem view helps identify activities, actors, and interactions in developing HR roles to capture the interdependencies, coordination and alignment that emerged at each level.

### 1.2. Human resource business partner role

Scholars have long argued about the importance of HR’s strategic role in aligning a company’s business objectives [16,47] and that HR should be considered a business partner to the company [48]. In 1997, Ulrich introduced a human resource business partner role, based on the study conducted by a sample of 256 HR professionals [49], comprised of strategic partners, administrative experts, employee champions, and change agents. This role is arguably the most popular framework describing HR roles to date. Each function with its own focus and unique expertise cannot be separated. HR is advised to execute the functions altogether, each with its own focus and unique expertise, to fulfil its responsibilities in delivering value to stakeholders [50]. As a company partner, HR must have a vision or strategy to prioritize people’s work and investments within goal achievement [50]. Furthermore, HR’s strategic role is apparent through its involvement in decision-making [20,51]. HR should develop its roles and execute its strategies and policies by collaborating with stakeholders to achieve this goal.

Previous research on the HR role was more on identifying the role the HR department has in the organization/company [52–54] and how the HR role influences other variables, such as business outcomes [15], HRM effectiveness [18] and organizational performance [21,55,56]. Through semi-structured interviews, the study of [52] define the difference between HR managers’ roles at the corporate and unit levels in the hospitality industry, while [53] was using a case study in a full-service bank, reported all four functions of Ulrich’s HR roles are presented. In Indonesia’s context, a survey conducted by [54] found that all four functions are practised by a state-owned oil and gas company, making them a business partner for the company. A study by [18] in Malaysia’s manufacturing companies confirmed the relationship between HR roles and HRM effectiveness through HR role stressors. HR role was also found to have an impact on organizational performance from the survey studies carried out by [21,55,56]. A study of [15] to Fortune 500 India companies found that HR roles mediates the digital HR technology to business outcomes.

## 2. Methodology

### 2.1. Qualitative phase

In this phase, Grounded Theory (GT) was employed for data analysis to explore the collaboration of actors and factors in developing HR role, as this study was the first step in using the ecosystem approach. A systematic process was used to use the GT approach to analyze the data from semi-structured interviews from the standpoint of HR specialists (manager and supervisor), which presented a perspective not previously supplied in study [57] (. Therefore, this study employed the GT techniques recommended by [58], whereby familiarity with previous research might increase sensitivity to the themes that emerge from the data [59]. Participants (see Table 1) were chosen through convenient sampling from various consumer goods companies. We collected data through ten interviews that lasted one hour (average) using video-conference meetings. To ensure data dependability and credibility, all interviews adhered to the same script, and questions were asked in the prescribed order [60]. The participants were asked questions about the HR role and how it developed. To prevent mistakes and misunderstandings, the interviews were recorded, transcribed, and approved by the participants [61].

**Table 1:** Overview of participants for the qualitative phase.

Demography	Category	Frequency	Percentage
Job position	Director	4	40%
	Manager	6	60%
Age group	31–40 years	4	40%
	41–50 years	5	50%
	> 50 years	1	10%
Gender	Female	5	50%
	Male	5	50%
Working Experience	6–10 years	4	40%
	11–20 years	4	40%
	21–30 years	2	20%

We then used the grounded theory method to evaluate the data [62]. By coding, categorising, and connecting data, this examination method, which largely relies on ongoing comparison of data gathering and analysis, enables the creation of inductive theories. Researchers used literature on the HR role and interview data in this procedure to compare the findings [63]. This continuous comparison involves two methods: open coding and axial coding. Initially, the data content was divided into main concepts or small sentences (elements); then finally they were inductively grouped according to their associations. The initial concepts of the developing conceptual model were able to be created through this procedure. Through iterative fine-tuning of the analytical structure and review of the data to seek confirmation of emerging themes, a conceptual model was assembled in which hypothesized interactive pathways were proposed based on the literature and content of the interview data.

### 2.2. Quantitative phase

In the quantitative phase, we tested the structure of the conceptual model developed. The constructs and measurement items were developed mainly from the literature on qualitative findings. A 64-item scale was devised to measure the managerial perspective of HRDE and HR role in their companies based on a 5-point Likert-type scale of 1 (strongly disagree) to 5 (strongly agree). All the measurement items are listed in Appendix A in S1 Appendix. We compiled a list of companies that satisfied the requirement for having an HR department. Through our relationship, we got in touch with them to discuss the goals of the study and gauge their enthusiasm for taking part. In the last phase, we acquired a random sample of consumer product companies. Only the invited companies had access to the questionnaire, which was completed by workers who held managerial roles and related staff at their respective companies (see Table 2). Due to the Covid-19 pandemic, the data were collected in two forms. The first one is through an online survey through google forms by sending the link address (June to December 2021) and through hardcopy questionnaires (October to December 2022) to reach more participants and generalizability. We asked the participants about human capital department roles and its development factors in their companies to complete the survey.

**Table 2:** Profile of respondents.

Demography	Category	Frequency	Percentage
Job Position	Human resource	46	18%
	Others	216	82%
Job Position	General Manager	2	1%
	Manager	44	17%
	Assistant Manager	46	18%
	Supervisor	145	55%
	Staff	20	8%
	Not answered	5	2%
Age Range	20–30 years	97	37%
	31–40 years	106	40%
	41–50 years	48	18%
	> 50 years	11	4%
Working Experience	0–5 years	115	44%
	6–10 years	73	28%
	11–20 years	56	21%
	21–30 years	16	6%
	> 30 years	2	1%
Gender	Female	94	36%
	Male	168	64%

We used structural equation modelling (SEM) in the current investigation to model hypotheses and evaluate novel causal links between constructs based on the conceptual framework that was proposed in the qualitative phase. PLS-SEM offers an appropriate statistical method for testing hypotheses in exploratory research in a way that is generalizable [64,65]. To evaluate the convergent validity of PLS-SEM, the average variance extracted (AVE) is proposed. For exploratory research, the AVE must be at least 0.5, composite reliability must be at least 0.7, and 0.6 is acceptable [66]. Additionally, factor loading values of 0.70 or greater are recommended, while 0.4 or greater are acceptable for exploratory research [67]. Additionally, by using the criterion, discriminant validity is assessed [68]. Each latent variable’s square root of AVE should be greater than the correlations among the latent variables for discriminant validity [68]. A preliminary structural model evaluation should consider the path coefficients and the R-square (R2) measure of endogenous constructs (i.e., inner model). R2 is highly reliant on the research area, however, the path coefficients must be significant [69], who claimed that R2 measurements of 0.67, 0.33, and 0.19 were significant, moderate, and weak, respectively.

For both phases, the study has met the requirements of the ethical research conducted at the School of Business and Management, Institut Teknologi Bandung (SBM ITB), and full ethical approval has been granted on behalf of the SBM ITB Research Ethics Committee by Dr. Anggara Wisesa. Moreover, all interviews’ participants and survey’s respondents have been given written informed consent before the interview was conducted and at the beginning of the survey form.

## Results

The results are divided into two distinct parts: the development of the conceptual model and hypotheses paths, followed by the test of the structural model of the quantitative phase.

### 3.1. Qualitative phase and hypotheses development

**Fig 1:**
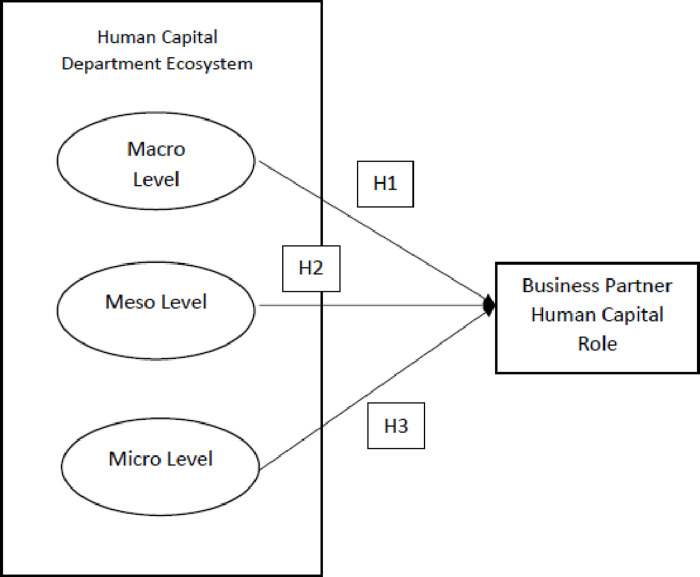
Research model.

Data content obtained from the ten interviews and analyzed with grounded theory, coded and grouped into three layers of the HRDE, comprised of eight actors and four factors (Tables 3 and 4) in developing HR role (Fig 1). Below, we analyze the actors and factors and propose hypotheses paths.

**Table 3:** The human resource department ecosystem’s actors.

Level of Ecosystem	Actors	Roles
The micro level of the Ecosystem	HC representative at the board	Collaborate with the top management to voice people’s agenda into the business decision-making process
The meso level of Ecosystem	Top management	Collaborate with HC as a strategic business partner to involve people’s strategy in the company’s business.
	Line manager	Collaborate with HC as a discussion partner to find people-related business solutions
The macro level of the Ecosystem	Outsourcing and Vendor	Collaborate with HC to support the administrative and strategic HC in daily activities
	Business and Professional Associations	Collaborate with HC by enhancing the knowledge and capability of HC professionals in terms of human capital, business, and policy knowledge
	Community	Collaborate with HC by supporting the HC activities in increasing the company’s image and engagement from the community
	Government	Collaborate with HC by supporting the HC activities in labor-related matters, mostly in industrial relations
	University	Collaborate with HC in supporting HC programs such as recruitment, training, internship, and knowledge development

**Table 4:** The human resource department ecosystem’s factors.

Level of Ecosystem	Factors	Roles
The micro level of the Ecosystem	HC skills and competencies	To support the collaboration between HC and other stakeholders
	HC structures	To support HC in prioritizing the HC business partner role
Meso level of Ecosystem	Adoption of Digital HC	To support the HC role administratively and strategically
	Company Culture	To support the role of HC involved in the business

#### 3.1.1. Macro layer

In developing the HR business partner role, it is found that the HR department made collaboration with some actors outside the company. The actors are grouped into 5 main actors, namely: outsourcing and vendor; business and professional associations; community; government; and university. Below are the explanations of each actor’s collaboration in detail. Collaboration with outsourcing companies and vendors (OV) is essential in supporting HR strategic and administrative roles. Interviewees confirmed that HR collaborates with OV in performing most administrative tasks, although external experts such as consultants in strategic planning were required. Administrative activities such as recruitment and payrolling are commonly outsourced, as explained below:

*“(We use) payroll vendors*, *training vendors*, *consultants*, *Organization Development consultants*, *and manpower outsourcing vendors who collaborate with us*.*”* (HR Director Company C)

HR also collaborates with training vendors and external experts, including consultants, lawyers, and other external partners, in performing strategic tasks. Although interviewees highlighted the importance of employee development through internal staff training programs, external coaching is regarded as providing additional knowledge. Learning from experts partly explains why training vendors constitute HR partners in developing employee capacity. Other vendors, such as those involved in recruitment, IT, and content & design, support HR’s daily activities. An external consultant supports strategic HR role in organizational development, employee performance assessment, talent management, and culture design implementation.

In performing HR-related activities, HR collaborates with businesses and professional associations to learn more about business acumen-associated competencies and HR-related matters, thereby executing the role of BP more effectively. Business associations offer support to HR professionals by increasing access to information about business acumen and enhancing their competencies, rendering them more competent BP. Professional association members, predominantly HR practitioners drawn from various companies, help deepen HR-related knowledge, including HR competencies and government regulations. A professional HR association enables HR professionals to meet and discuss important issues. Certain interviewees became active HR community members, sharing relevant knowledge with fellow professionals and academics. Furthermore, HR leaders view professional associations as community networks serving HR issues platforms.

The community is those individuals living near the company site whose presence impacts them socially. To achieve a mutually advantageous balance of interests, the company supports the local community by hiring those members with appropriate skills while simultaneously creating a mutually positive working atmosphere. By promoting and maintaining effective cooperation with the community, HR helps the company conduct its business as a form of BP. To intensify local community engagement with the company, HR is often involved in its activities such as corporate social responsibility (CSR) programs or marketing fairs.

HR departments have also cooperated with such universities to support their developed programs, including recent graduate recruitment, internships, guest lecturers, and expert-led consultancies intended to increase company branding and campus engagement. HR forges links with particular universities to attract new talent satisfying specific criteria through events such as job fairs. Training programs and research from the such collaboration are intended to promote academic knowledge acquisition. Moreover, HR practitioners value the insights of academics regarding HR theories that can be implemented by the industry, subsequently enhancing industry-academic cooperation and improving industrial practices. Such mutually beneficial collaboration renders HR an SP for the company.

The final actor is the government through its Office of Manpower, which handles labour-related or employee issues. Most involve industrial relations, which fall within HR’s EC role of focusing on workers’ needs. Government-regulated industrial relations are indispensable to the manufacturing sector because they manage the relationship between companies, employees, trade unions, and the role of HR as a BP. Within industrial relations, HR works together with lawyers who review government-mandated documents. Another collaborative activity is that of supporting the government in providing apprenticeships.

We know from previous studies [16,26] that the external factors influenced the HR role that, in fast-changing business competitiveness, it is required to expand the mutual collaboration with as many actors as possible including those outside the company. The involvement of outsourcing and vendors in the company’s HR activities is crucial in helping HR to do some administrative and strategic business. Relation and collaboration of the HR department with business and professional associations enhance HR’s knowledge and capability in terms of human capital, business, and policy knowledge in applying for its business partner role. The government gave a guideline for HR in managing people as an asset of the company, mostly in industrial relations. The collaboration with the university in forms of research as well as other HR activities such as recruitment, training, internship, and knowledge development to enhance the HR business partner role, especially in the employee champion function. As for the community, the collaboration supports the HR activities in increasing the company’s image and engagement from the community.

[26,70], and [71] are a few studies that have previously discussed the role of the external environment in supporting HC roles. Using both internal and external mechanisms, [71] claims that human capital can be developed through collaboration. Three external stakeholders related to HC are listed among the five key stakeholders mentioned by [26]: employee, line manager, customer, investor, and community. Investment from HC should match customer expectations if you want to boost customer engagement. When developing HC investments, it’s also important to take into account boosting investor confidence and the company’s reputation in the neighborhood [26,70] mention the small labor market, government meddling, other higher authorities, and the labor unions as the outside variables affecting the HC role. In the HC ecosystem, the external stakeholders support the role of the HC through cooperation with the HC department. This study identified the external stakeholders at the macro level to be defined. The macro level of an ecosystem is comprised of vendors and outsourcing, business and professional associations, academia, government, and community. The results of our initial phase demonstrate support for external stakeholders, which are categorized into an ecosystem’s macro level. The collaboration between the external stakeholders and the HC department shapes HC’s role in the eyes of the outside world. On the basis of the arguments above, we formulated the hypotheses that:

Hypothesis 1 (H1): The macro layer, including outsourcing and vendor; business and professional associations; community; government; and university; positively influence the development of HR business partner role.

#### 3.1.2. Meso layer

Inside the company, the HR department collaborated with top management and line managers, to assist them with the people strategy in their business decisions. This collaboration made the HR role becomes an important business partner. Some factors inside the company are also crucial in developing the role: company culture and adopting an HR information technology (IT) system. The top management (TM), whose support and trust of HR leaders, are significant for HR in implementing its initiatives and activities, and achieving individuals’ business-related agendas while also providing support and delegating the HR-related issues to be handled by HR. TM needs HR to provide input on relevant issues and analysis to support its decisions, such as when seeking to expand its product range or target markets and when confronted by novel work concepts which need HR as a CA and mediator between employer and employees. This collaboration becomes vital in developing HR’s role as a company’s BP and CA.

The second actor is the line manager (LM), a term representing the business leaders of other functions. Positive interaction and collaboration with other business leaders are required to develop an HR BP. HR needs the LM to be fully responsible for employees under his/her supervision and LM needs HR to be an expert in personnel issues and an active partner in discussions about people-related business decisions. The Human Resource Business Partner (HRBP) position addresses this need. As an HR representative for each function, HRBP assists business leaders in ensuring effective communication between HR and LM. Collaboration between HR and business leaders enables HR to be involved in business decisions through each commercial function, rendering HR a strategic BP.

The adoption of digital HR and company culture are supporting factors underpinning the HR role. Digitalization in the HR sphere supports many HR-related jobs to become easier and more rapidly executed. Adopting this digital HR supports the provision of administrative services to employees and renders HR more strategic by using data analysis as part of the HR-related business decision-making process. The adoption of digital HR, such as HRIS, reduces the administrative load and enables HR to focus more on its strategic role. The participating companies appear to use digital HR records extensively in the form of HRIS, while other digital platforms’ chatbots facilitate HR provision of feedback on employees’ routine problems.

The prevailing company culture constitutes the next supporting factor HR’s role can be well-developed where the company culture, initially shaped by TM and subsequently disseminated by the HR CA, supports it. Discussion of the strategic role of HR as a company’s BP must embrace how that company views the importance of its employees’ agenda. This situation encompasses how a company perceives the importance of HR; how its structure is formed; how its leader is involved in the business decision-making process as a representative on the board; and, lastly, how the employees’ agenda aligns with business objectives in forming part of the company’s culture. The latter’s importance in shaping HR’s role can be seen in the previous discussion on how HR leaders participate in boardroom discussions and the decision-making process. How the company forms its HR structure includes the presence of an HR representative on the board. HR can participate strategically in a company’s commercial development rather than merely executing administrative tasks.

The adoption of digital HC, top management support, line manager expectations of the HC role, and the significance of culture and values are all grouped into the meso level of an ecosystem in developing HC roles. In previous studies, it has been contested that top management should assist HC in carrying out tasks related to HC. In their research, [72] discovered that the CEO/chief executive plays a crucial part in delivering HC capability. CEO perspectives were used to determine the role of HC in putting into place a more efficient HC system in a qualitative study by [73]. Additionally, the CEO must be willing to delegate duties associated with HC-related activities, particularly to the HC department, and possess a broader understanding of HC activities.

According to a study by [74], the effectiveness of the HC department should be increased by utilizing the relationship with line managers. From the interviewees’ statements during the qualitative phase, the line manager serves as the unit’s HC manager. The line manager’s involvement in creating HC roles depends on the input the HC department could provide regarding HC-related decisions. The HCBP should be involved in decisions involving human capital [75]. In addition to acting as a liaison between the line manager and the human resources division, the role of HC Business Partner also functions as an internal human resources consultant for a business unit. The HC department must live up to the expectations of the line manager if they are to be more involved in HC-related decisions and activities. Previous studies [17,76] have discussed the use of information systems as a tool for improving digital HC. Findings from the first phase also highlight how adopting digital HC in developing HC roles becomes important, not just in an administrative role but also in employee champion and strategic HC roles.

HC roles also emerged in the organization, where HC is viewed as a strategic partner that aids in making business decisions. The company’s culture and values are what create this environment. The study of [17,77,78] is thus extended based on the results of the first phase of this research to incorporate corporate values and culture into the development of HC roles. From those elaborations and in line with previous works, we formulated that:

**Hypothesis 2 (H2)**: The meso layer, which consists of top management; line manager; company culture; and adoption of HR IT system; positively influence the development of HR business partner role

#### 3.1.3. Micro layer

The micro layer is located inside the HR department. The role of the HR department leader, moreover as a representative at the board, is also essential in developing the HR business partner role. Other factors are the competencies of HR manpower as assets of the company. Moreover, the structure of the HR department defined the role HR wants to show. Below is the explanation of the findings. As a representative on the board, the HR leader’s role is to escalate HR issues related to business strategy. The direct line of reporting from the HR leader to TM and their collaboration ensures HR an equal voice as other functions in solving any business-related problems. The interaction and collaboration between HR leaders and TM involve HR in companies’ strategic decisions. The HR leader helps TM arrive at business decisions by escalating and discussing HR issues affecting the enterprise. The HR issue becomes part of the business strategy and decision-making process, which is reflected in the following informant’s comment:

*“In every discussion about the company strategy*, *I am always involved*, *and there is always a strategy regarding people*, *organization*, *capability*, *and culture*. *Therefore*, *it is impossible; our chairman never discusses a strategy without an HR representative present*. *There always will be*, *and HR will always be part of the strategy”*. (HR Director Company B)

Two supporting factors are important in determining HR roles; relevant skills and competencies and HR structure. Several competencies mentioned are HR business credibility, HR expertise, and general competency. HR business credibility is mentioned as an important competency to be mastered in which HR should be SP of the business. HR business credibility enables them to have conversations with other business leaders on an equal footing and fruitful discussions with other business functions in determining people-focused commercial decisions. HR must also master HR expertise competencies to provide solutions to business leaders from HR’s perspective. HR should be competent in essential competencies ranging from recruitment, compensation, benefit, and employee development to employee termination. Competency mastery enables HR to provide inputs into any HR-related matter as requested by business leaders. Effective communication skills are also necessary for HR to communicate the importance of people’s commercial agendas.

Another supporting factor was the HR structure, the HR department’s organizational framework formed by the company to provide premium services to its stakeholders. Participants’ companies formed strategic divisions to perform HR roles by having appropriate expertise or a centre of excellence (COE), the brains behind every HR-related policy and regulation, as a BP. There is also an organization development division that matches business needs to how HR would prefer to respond as an organization. HR administration and shared services such as payroll and benefits play a more administrative role for employees. The HR department structure reflects the importance of HR as a strategic BP of the company. By dividing the HR structure into strategic areas such as organizational development, employee development, industrial relations, and administration (including employee support), HR can implement every strategic and administrative decision. Another strategic role is manifested by the post of HRBP, which reflects the company’s focus on its strategic HR role.

As a result of the first phase’s findings, the development of human capital roles was influenced by factors at the micro-level of an ecosystem, including HC structure, HC skills and competencies, and HC representation on the board. According to a study by [75], the company transformed its HC structure by designating human capital business partners to the business unit in order to strengthen the HC department’s ability to be more strategic in managerial decisions and add value to HC activities. The goal is to improve communication between the business unit and the human capital department. [50] also describe the organizational structure of the HC department, which ought to function as a business inside a business to add value and develop into a strategic partner for the business. According to the study, the HC division was broken down into service centers, corporate HC, embedded HC or HC business partners, centers of expertise, and operational executors. In the initial phase, it was discovered that businesses divided the HC activities into a number of administrative and strategic divisions. The HC department can perform its duties and play a more strategic partner in business thanks to this structure.

The development of HC is also influenced by HC skills and competencies. To maximize the effectiveness of HC professionals, it’s critical to master the fundamentals of the field and comprehend the business environment as a whole [79]. Having a HC representative on the board allows the business to include HC activities in all business decisions, which strengthens the role of HC. [80] also speak about the elevated position of HC leaders that CEO success should be more dependent on HC leaders.

It is the responsibility of HC leaders to forecast results, identify issues, recommend actions that will add value, and advise on what not to do in HC-related business decisions. [80] suggested that the CEO should work more closely with the CFO and CHCO to form a "triumvirate at the top of the corporation" in order to turn the HC leader into a genuine business strategic partner. Given the above arguments, we hypothesized that:

**Hypothesis 3 (H3):** The micro layer, which comprises of HR leader as a representative at the board; HR staff competencies; and HR department structure; positively influence the development of HR business partner role.

### 3.2. Quantitative phase

#### 3.2.1. Measurement and structural model

In order to examine the reliability and validity of constructs in the model, an evaluation of the measurement model was conducted. To test the measurement and structural models, employees in managerial positions and related staff, either holding an HR role or being accountable for HR-related duties, in consumer goods companies were issued an online questionnaire using a google form to administer the instrument, which was based on the qualitative findings. The final sample we obtained was 262 legitimate responses, and the profiles of the respondents are shown in Table II. Since the structural model can only have three arrows pointing at any given construct, we built an acceptable model and determined that 176 samples would be sufficient to detect a minimal R2 value of 0.10 in any of the constructions at a significance level of 1%. [81]. We met the sample size criteria because we had 262 viable responses.

Factor loadings and average variance extracted (AVE) were the basis for examining the convergent validity of the measurement model. Initially, 64 measurement scales were used to investigate the construct’s reliability and validity. However, one item with an outer loading of less than 0.4 was excluded from further analysis. Most factor loading values of all the components were higher than 0.4, representing ’satisfactory to good’ reliability levels. The AVE values of the constructs, ranging from 0.521 to 0.575, were higher than the recommended value of 0.5. All constructs’ composite reliability (CR) values ranged from 0.813 to 0.966, higher than the recommended value. Table 5 presents CR, AVE and factor loadings. The results outlined above confirm the convergence validity of the measurement model. Discriminant validity was checked using the Fornell-Larcker criterion. To satisfy the requirements, another 26 items were excluded from further analysis. The square root of AVE for each construct had the highest value compared to other correlation values, showing a relationship with other factors. The results satisfy the requirements and empirically validate the suitability of the measurement model employed in this study.

**Table 5:** Result of construct validity and reliability.

Construct	Item	Factor Loading	CR	AVE
Micro layer of ecosystem (Micro)	MI1	0.762	0.871	0.575
	MI3	0.769		
	MI4	0.761		
	MI6	0.805		
	MI7	0.691		
Meso layer of ecosystem (Meso)	ME1	0.743	0.893	0.544
	ME2	0.774		
	ME3	0.727		
	ME5	0.71		
	ME7	0.759		
	ME8	0.721		
	ME9	0.726		
Macro layer of ecosystem (Macro)	MA1	0.733	0.813	0.521
	MA2	0.762		
	MA3	0.642		
	MA4	0.745		
Human Resource Role (HRR)	HRR6	0.698	0.966	0.561
	HRR8	0.772		
	HRR9	0.733		
	HRR10	0.732		
	HRR11	0.759		
	HRR12	0.738		
	HRR13	0.746		
	HRR14	0.789		
	HRR16	0.754		
	HRR17	0.793		
	HRR19	0.726		
	HRR22	0.721		
	HRR27	0.68		
	HRR31	0.703		
	HRR32	0.747		
	HRR33	0.739		
	HRR34	0.717		
	HRR36	0.791		
	HRR37	0.785		
	HRR38	0.812		
	HRR39	0.766		
	HRR40	0.765		

A structural model was used to determine the goodness of fit statistics of the proposed theoretical structure. The coefficient of determination (R^2^ value) tests a model’s predictive accuracy and relevance [81]. The R^2^ values of the Human Resource Role (HRR) construct were 0.654, indicating a substantial level of predictive accuracy. Moreover, the blindfolding procedure calculated the Q^2^ value to evaluate predictive relevance. The value of Q2 > 0.05 confirmed the predictive relevance among endogenous variables in the model [81]. The statistical results showed that the Q^2^ values produced for HRR is 0.362, were positive, implying the adequate predictive relevance of the proposed model in this study. Moreover, the analysis generated the goodness of fit (GoF) result by multiplying the square root of the average value of R^2^ and the average value of Q^2^, which ranges from 0 to 1 [82,83]. The GoF result was 0.293, indicating that the model can explain the empirical data.

#### 3.2.2. Hypothesis testing

Three hypotheses positing direct relationships between the constructs were empirically supported (see Table 6). The T-values were more than 1.96 and P. Values < 0.05. It can be concluded that Hypothesis 1 was supported (ß = 0.217, T = 4.501, P = 0.000) and that the macro has a significant positive effect on the HRR. Hypothesis 2, that meso has a significant positive effect on the HRR, was supported (ß = 0.284, T = 4.893, P = 0.000). Hypothesis 3, that micro has a significant positive effect on HRR was supported (ß = 0.411, T = 6.784, P = 0.000). These results indicated that each level of the HRDE (micro, meso, and macro) has significant effect in developing HR roles as well as confirming the results from the qualitative phase.

**Table 6:** Results of the hypothesis testing.

Hypothesis	Hypothesized path	T Statistics	Effect	P Values	Decision
H1	Macro -> HRR	4.501	0.217	0.000	Supported
H2	Meso -> HRR	4.893	0.284	0.000	Supported
H3	Micro -> HRR	6.784	0.411	0.000	Supported

## 4. Discussion

We emphasise that established processes were considered when developing and validating the conceptual framework. In the qualitative stage, experts from consumer goods firms were chosen as HR professionals. The conceptual framework was then developed after data were analysed using grounded theory. In the quantitative step, we examined consumer goods businesses that had human resources divisions. Finally, we performed the data analysis methods. Our findings confirm that these actors and factors offer a comprehensive strategy for fostering the growth of the HR role. According to [67], the model structures statistically explain 65,4% of the HRDE in developing HR roles, which is a noteworthy finding and is considered a moderate result.

We explored the collaboration of the HR department with actors in developing HR roles and examined the relationship between each ecosystem level in developing HR roles. We found that eight actors and four factors are involved in developing the HR role following initial exploration. The HR department mutually interacts with each ecosystem actor. From the micro, the collaboration between the HR leader as a representation of the board and the TM team can be seen from the involvement of HR issues in strategic decisions. From the meso, TM collaborates with the HR department by providing support and trust to HR in undertaking HR-related activities supporting the business. Meanwhile, the HR department and LM collaboration are shown by supporting the LM in making HR-related business decisions. From the macro, OV assists the HR department in performing administrative and strategic tasks.

The interactions between HR and business and professional associations enhance the former’s business acumen and competencies. HR also assists the company in providing mutual benefits to the community through the recruitment program. Meanwhile, the collaboration between HR and the university supports an industry-academic cooperation ethos. Finally, cooperation between the HR and the Office of Manpower signifies a partnership between the parties. From the hypothesis testing, we found that the micro, meso and macro significantly affect the HR role.

The qualitative and quantitative findings are consistent with the internal and external factors involved in performing HR role. At micro, for instance, [84] found that the strategic HR role is indicated by the appointment of HR directors to TM teams. [50] emphasized the significance of the HR structure in reflecting its needs. [17,22,73] highlighted the importance of HR professionals’ competency as a factor in strengthening HR roles. Meanwhile, when examining meso, studies by [22,85] and [71] state that TM support is vital in developing HR roles. LM is also often discussed concerning HR [15,17,25,85] conclude that HR transformation could possibly be done using the digital HR technology and through HR roles, while [76] posit that the information system supports HR administrative roles but not strategic ones. [78] and [17] are among the researchers who highlight the importance of company culture in shaping HR roles. The collaboration between two actors, the top management and the line manager, with the HR department, is supported by the adoption of an HR information system and company culture.

Less research on external environment to HR role that some previous studies demonstrated that, in collaboration with outsourcing and vendor, business and professional associations, communities, universities, and government constitute the external environment in developing HR role. This collaboration, in which external consultants and outsourced providers are part of HR work, is mentioned by [24], while [86] discussed the collaboration with society and other organizations as external stakeholders. [87] analyzes the government’s role in implementing policies and enacting laws relating to HR’s work, leading to a discussion of HR as a profession. [88] review the sharing of HR activities among agents, including outsourcing providers as external agents. [26] also mention communities as HR stakeholders. Furthermore, HR’s involvement in community service enhances a company’s reputation within the community. Collaboration with universities is required for recruitment [71].

Other researchers have theorized the significance of collaboration and the ecosystem perspective in HRM. [37] proposed the workforce ecosystem; [38] discussed the HRM ecosystem in contemporary organizing; while [34] explore the importance of an ecosystem approach to HRM in a gig economy. Our study expands on the previous research into HR role by using an ecosystem approach in analyzing the collaborating actors and the factors supporting HR role development. The HR department is a unit with complex activities that the whole ecosystem should support in developing its role as a BP for the company. By having the whole ecosystem supporting its role as a business partner, the HR department could maximize its role in developing the employees in the company. BP’s inseparable four roles, SP, CA, AE, and EC contribute to employees’ development. SP contributes to how employees are a vital asset for the company’s business; CA contributes to the better alignment of the company’s strategy into the development of employees; AE contributes to the better of employees’ administration related to HR matters; while EC contributes to the need of the employees especially in upgrading employees’ competencies in business acumen and HR issues, which related to employees’ confidence level to take part in the business strategy [11].

## Conclusion

This study investigated and evaluated the interactions and contributions to the evolution of the HR role made by actors and factors in each layer of the ecosystem, including the internal HR department, internal business, and external business. This study provided a theoretical response to the paucity of literature on the evolution of HR roles. By addressing key players and elements of HRDE in creating HR roles, also advances theory and practice. This paper expresses its authors’ conviction that an ecosystem perspective should be adopted when analyzing and developing HR roles since it enables HR proponents to collaborate with the necessary number of stakeholders in developing the roles and assisting organizations in redefining the importance of business-related HR roles. We hope that the ideas presented provide a starting point for further investigation of HR roles and that future research will explore another critical stakeholder in supporting HR role development and how stakeholders shape HR roles.

### Theoretical contributions

There is a need for greater research on the many aspects of establishing HR roles because there is little existing research on the subject. Our research adds to the limited body of literature on the development of HR roles through actor/stakeholder interaction [25,89] by embracing systems thinking [86] by proposing the ecosystem perspective. Additionally, we take a fresh approach to role development by using an ecosystem approach. The conceptual model developed and tested in this study represents original research in which we adopted a mixed-method approach and conducted rigorous analysis (grounded theory and PLS-SEM) on the data collected to ensure substantial implications. Thus, we present the theoretical and managerial implications of this study.

The main contribution is the proposition and test of a conceptual model, including the relationship between each human resource department ecosystem level in developing human resource role. First, eight actors and four factors were inductively grouped in the three levels of the human resource department ecosystem. Then, by putting the model to the test, we can statistically extrapolate how they relate to one another, which is a big addition since it exposes how important each one is in relation to the others. This outcome so supported the qualitative and quantitative approaches we used to conduct the study and respond to the research question.

This is one of the first empirical studies to use a mixed-method approach to investigate the interdependencies between the HR department and the ecosystem’s characteristics at all layers of the ecosystem in developing HR roles, whereas previous research on the ecosystem theme in HRM is conceptual in nature. The ecosystem’s actors, activities, and interactions were interpreted based on the data produced by semi-structured interviews. This qualitative study bridges the research gap by analyzing the symbiotic relationship between those actors and the HR department as an ecosystem.

Our study highlights the potential value of the ecosystem approach in developing HR roles which should focus on HR research [12] through the interdependencies and collaboration of ecosystem actors and supporting factors. We suggest that by identifying stakeholders and factors supporting HR role development at three ecosystem layers, based on the existing position within the company, each collaborating actor participates in developing HR roles as a BP for the company. The HR head and staff collaborate with TM, LM, and external parties to make HR a strategic BP.

### Managerial implications

The HR BP’s role is considered essential for a company. However, the development of this role has been discussed separately. This study provides a new perspective on how this role is developed through the lens of the ecosystem approach by assessing three ecosystem characteristics. Through this approach, interdependencies between actors and the HR department can be viewed holistically, rendering the HR BP role as the value creation of the HR department. This research also highlights how the HR department could be more effectively and valuably involved with all stakeholders in developing BP roles.

The results obtained led to the proposition of a framework in which the HR department has a symbiotic relationship with all actors across the ecosystem, playing an essential role in developing the role of HR. Eight actors collaborating to support HR role development were identified with each activity and interaction. These findings enable the development of a framework to assist company leaders in understanding the implications of stakeholders’ interdependencies, from a multi-layer perspective of the ecosystem, in terms of impacting the HR department’s strategies and performance when executing their BP roles. In fact, despite the numerous contributions acknowledged in the existing research, a framework for exploring stakeholders’ development of HRR as BP from an ecosystem perspective remains to be proposed.

The findings, however, have significant implications for HR, particularly HR role. This study will support TM, HR leaders, and practitioners in defining, designing, and positioning HR roles within companies. A BP role is crucial for the company to align business strategies with individuals’ agendas. These findings suggest that stakeholder collaboration and certain factors at each ecosystem level determine HR role development. The HR department should collaborate with as many internal and external stakeholders as possible while also reviewing the factors supporting the development of HR role.

The study can make some contributions to practice in terms of its management implications, which mostly focus on how businesses might use their efforts to improve HR roles. First, managers may get the overall picture of the aspects they should be conscious of when selecting how to develop the HR role by using the developed model. Second, we advise practitioners to pay more attention to comprehending the ecosystem’s external environment in addition to its internal ecosystem layer. Understanding how external players contribute to the ecosystem’s success is crucial for boosting the ecosystem’s resilience. Building long-lasting, trust-based relationships with ecosystem partners is made simpler by knowing their roles and effects [90] Finally, we suggest that outside parties including businesses, governments, and educational institutions educate today’s ecosystem orchestrators and partners. As opposed to conventional (internally focused) corporate management, ecosystem orchestration and participation demand a distinct mentality.

### Limitations and future research

The limitations of this study should be considered when drawing any findings because they open up possibilities for additional research. First of all, this study’s primary focus was on expanding HR roles through the HR department ecosystem. It was conducted through interviews with HR specialists in the consumer goods sector. As a result, it probably does not include all the important players and factors that might be present. As a result, the present study could have benefited from conducting more interviews with participants from different industries, as this would have provided richer data and the chance to explore more in-depth themes. Second, in this study, the key informants were HR leaders whose understanding and perceptions of HR’s role, although necessary, do not fully represent all stakeholders’ perspectives. A broader stakeholder view would promote a more comprehensive understanding of HR roles and their involvement in development. Further research is required to expand the boundaries of this analysis to other populations.

## Supporting information

S1 Appendix Appendix A. constructs/items used in the questionnaire. (DOCX)
