# Supplementary material for: Dynamics of human resource department ecosystem in developing human resource role: An ecosystem perspective
Source: PLoS One. 2023 Dec 14;18(12):e0295544. doi: 10.1371/journal.pone.0295544 (PMC10723775; doi:10.1371/journal.pone.0295544)
Supplement: S1 Appendix — (DOCX) [file pone.0295544.s001.docx]

**Appendix A. Constructs/Items Used in the Questionnaire.**

| Construct | Definition | Item | | Adapted from |
| --- | --- | --- | --- | --- |
| Micro level of ecosystem (MI) | An ecosystem level in developing HRD roles, which scope is limited within the human capital department. The micro level of the ecosystem consists of the skill and competency of HR professionals and the characteristics of the HR department. | MI1 | The HR department leader has a compelling vision of the methods of using HR to improve company performance by developing a well-understood HR strategy for the company. | Khatri et al. (2016) |
|  |  | MI2 | The HR department leader has an understanding of the basics of business delivery procedures in the consumer goods industry and the distinct operating attributes of all the departments in the company. |  |
|  |  | MI3 | HR workers possess complete skills in HR and form efficacious in-house instruments, such as relevant recruitment approaches, functional training programmes, and equitable and conducive reward systems). |  |
|  |  | MI4 | HR employees have a comprehensive understanding of the approach, culture, and activities of the organisation. |  |
|  |  | MI5 | The basic measure is turned into a ratio of the number of HR professionals to the number of employees. | Valverde, et al. (2006) |
|  |  | MI6 | The human resource department has an organisational structure that is divided into strategic activities and operational activities. |  |
|  |  | MI7 | The HR leader is a crucial part of the top management team. |  |
| Meso level of ecosystem (ME) | An ecosystem level in developing HRD roles, which scope is limited within the company. The meso level of the ecosystem consists of top management support, line managers’ expectation, and internal factors of the company. | ME1 | The CEO views the HR function as an important part of the implementation of strategic actions of the organisation. | Khatri, et al. (2016) |
|  |  | ME2 | The company CEO completely acknowledges the crucial part played by HR in business delivery. |  |
|  |  | ME3 | The CEO offers full resources and support to HR programmes and operations. |  |
|  |  | ME4 | The department is capable of impacting the results of primary decisions in the business. | Teo and Rodwell (2007) in Nguyen and Teo (2018) |
|  |  | ME5 | The HR department fulfils expectations in the parts played by HRM. |  |
|  |  | ME6 | This organisation is an entrepreneurial place with a high dynamic, in which the dedication to innovation establishes a connection between the organisations. | Quinn and Spreitzer (2001) and Wei et al. (2008) |
|  |  | ME7 | This organisation places emphasis on the development and acquisition of new resources. |  |
|  |  | ME8 | A comprehensive use of information technology is applied, which sustains the administrative functions in the human resource field. | Haines and Lafleur (2008) |
|  |  | ME9 | Overall, information technology sustains the strategic functions of the human resource field, which is employed as analysis material to make decisions. |  |
| Macro level of ecosystem (MA) | An ecosystem level in developing HRD roles, which has broad scope outside the company. The macro level of the ecosystem consists of networking with the government, academia, other businesses, and the community. | MA1 | There are government regulations that sustain the regulation of the parts played by the human resource department. | Galvao, et al. (2017) |
|  |  | MA2 | The government has human resources programmes that support the part played by the human resource department and professionals in it. |  |
|  |  | MA3 | There is a curriculum that focuses on shaping and managing the part played by the human resource department in higher education, such as vocational schools, colleges, and business schools. |  |
|  |  | MA4 | The results of research and development performed by academics are useful for human resource department operations. |  |
|  |  | MA5 | There is a collaboration between the HR department with other businesses, such as outsourcing companies, vendors, and external experts to support HR department activities. |  |
|  |  | MA6 | There is financial support from other institutions aside from educational institutions for human capital professionals to develop their skills and competencies. |  |
|  |  | MA7 | There is a collaboration with the community or society in reinforcing the part played by the human capital department. |  |
|  |  | MA8 | Cooperation is present between business associations and professional associations in the field of human resources. |  |
| Human Resource Role (HRR) |  | HRR1 | HR helps the organisation accomplish business objectives. | Mamman and Somantri (2014) |
|  |  | HRR2 | HR is involved in the procedure of determining business approaches. |  |
|  |  | HRR3 | HR ensures the correspondence of HR approaches with a business approach. |  |
|  |  | HRR4 | HR efficacy is indicated through its capability to ensure the functionality of the approach. |  |
|  |  | HRR5 | HR is perceived as a business partner. |  |
|  |  | HRR6 | Strategic concerns involve time consumption for HR. |  |
|  |  | HRR7 | HR is actively involved in business scheming. |  |
|  |  | HRR8 | HR functions in aligning HR approaches and business approaches. |  |
|  |  | HRR9 | HR establishes programmes and procedures to connect HR approaches to gain business approach. |  |
|  |  | HRR10 | HR's reliability originates from the assistance in fulfilling strategic objectives. |  |
|  |  | HRR11 | HR makes further improvements in the company operating efficacy. |  |
|  |  | HRR12 | HR is involved in the delivery of HR procedures. |  |
|  |  | HRR13 | HR ensures the efficient administration of HR procedures. |  |
|  |  | HRR14 | HR efficacy is indicated through its capability of systematically delivering HR procedures. |  |
|  |  | HRR15 | HR is perceived as an administrative expert. |  |
|  |  | HRR16 | Operational concerns involve time consumption for HR. |  |
|  |  | HRR17 | HR is actively involved in constructing and executing HR procedures. |  |
|  |  | HRR18 | HR functions in monitoring administrative procedures. |  |
|  |  | HRR19 | HR creates programmes and procedures for the efficient processing of transactions and documents. |  |
|  |  | HRR20 | HR reliability originates from enhanced productivity. |  |
|  |  | HRR21 | HR assists the company in managing the workers' personal needs. |  |
|  |  | HRR22 | HR is involved in the improvement of workers’ dedication. |  |
|  |  | HRR23 | HR ensures that HR programmes and policies show responses to workers’ personal needs. |  |
|  |  | HRR24 | The measurement of HR efficacy is conducted in terms of its capability of helping workers fulfil personal needs. |  |
|  |  | HRR25 | HR is perceived as an ace for the workers. |  |
|  |  | HRR26 | HR’s time is dedicated to paying attention to and providing responses to workers. |  |
|  |  | HRR27 | HR is actively involved in paying attention to and providing responses to workers. |  |
|  |  | HRR28 | HR functions in assisting workers in fulfilling family and personal needs. |  |
|  |  | HRR29 | HR establishes programmes and procedures to manage workers’ personal needs. |  |
|  |  | HRR30 | HR reliability originates from the assistance for workers to fulfil their personal needs. |  |
|  |  | HRR31 | HR assists the company in adapting to change. |  |
|  |  | HRR32 | HR is involved in forming culture change for renewal and evolution. |  |
|  |  | HRR33 | HR ensures that its programmes and procedures enhance the company capability for changes. |  |
|  |  | HRR34 | HR efficacy is determined by its capability of assisting in a company prediction and adaptability to upcoming concerns. |  |
|  |  | HRR35 | HR is perceived as an agent of change. |  |
|  |  | HRR36 | HR dedicates its time to support emerging conducts for maintaining the company competitiveness. |  |
|  |  | HRR37 | HR is actively involved in the renewal, development, or evolution of the company. |  |
|  |  | HRR38 | HR functions in reshaping the conduct for organisational development. |  |
|  |  | HRR39 | HR creates programmes and procedures to assist in the company transformation. |  |
|  |  | HRR40 | HR reliability originates from creating changes. |  |

# References

[1]Teo STT, Rodwell JJ. To be strategic in the new public sector, HR must remember operational activities. Human Resource Management. 2007;46(2): 265-284.

[2]Quinn RE, Spreitzer GM. The psychometrics of the competing values culture instrument and an analysis of the impact of corporate culture on quality of life. In Woodman RW, Pasmore WA, editors. Research in organizational change and development. Greenwich, CT.: JAI Press; 2001;5. pp. 115–142.

[3]Wei LQ, Liu J, Zhang Y, Chiu, RK. The role of corporate culture in the process of strategic human resource management: evidence from Chinese enterprises. Human Resource Management. 2008;47(4): 777-794.

[4]Haines III VY, Lafleur G. Information technology usage and human resource roles and effectiveness. Human Resource Management. 2008;47(3): 525-540.

[5]Galvao A, Mascarenhas C, Rodrigues RG, Marques CS, Leal CT. A quadruple helix model of entrepreneurship, innovation and stages of economic development. Review of International Business and Strategy. 2017;27(2): 261-282.
